# T Cell Immunosenescence after Early Life Adversity: Association with Cytomegalovirus Infection

**DOI:** 10.3389/fimmu.2017.01263

**Published:** 2017-10-17

**Authors:** Martha M. C. Elwenspoek, Krystel Sias, Xenia Hengesch, Violetta K. Schaan, Fleur A. D. Leenen, Philipp Adams, Sophie B. Mériaux, Stephanie Schmitz, Fanny Bonnemberger, Anouk Ewen, Hartmut Schächinger, Claus Vögele, Claude P. Muller, Jonathan D. Turner

**Affiliations:** ^1^Department of Infection and Immunity, Luxembourg Institute of Health, Luxembourg City, Luxembourg; ^2^Department of Immunology, Research Institute of Psychobiology, University of Trier, Trier, Germany; ^3^Department of Clinical Psychophysiology, Institute of Psychobiology, University of Trier, Trier, Germany; ^4^Institute for Health and Behaviour, Research Unit INSIDE, University of Luxembourg, Luxembourg City, Luxembourg

**Keywords:** early life adversity, immunosenescence, CD57, cytomegalovirus, telomere length, T cells

## Abstract

Early life adversity (ELA) increases the risk for multiple age-related diseases, such as diabetes type 2 and cardiovascular disease. As prevalence is high, ELA poses a major and global public health problem. Immunosenescence, or aging of the immune system, has been proposed to underlie the association between ELA and long-term health consequences. However, it is unclear what drives ELA-associated immunosenescence and which cells are primarily affected. We investigated different biomarkers of immunosenescence in a healthy subset of the EpiPath cohort. Participants were either parent-reared (Ctrl, *n* = 59) or had experienced separation from their parents in early childhood and were subsequently adopted (ELA, *n* = 18). No difference was observed in telomere length or in methylation levels of age-related CpGs in whole blood, containing a heterogeneous mixture of immune cells. However, when specifically investigating T cells, we found a higher expression of senescence markers (CD57) in ELA. In addition, senescent T cells (CD57^+^) in ELA had an increased cytolytic potential compared to senescent cells in controls. With a mediation analysis we demonstrated that cytomegalovirus (CMV) infection, which is an important driving force of immunosenescence, largely accounted for elevated CD57 expression observed in ELA. Leukocyte telomere length may obscure cell-specific immunosenescence; here, we demonstrated that the use of cell surface markers of senescence can be more informative. Our data suggest that ELA may increase the risk of CMV infection in early childhood, thereby mediating the effect of ELA on T cell-specific immunosenescence. Thus, future studies should include CMV as a confounder or selectively investigate CMV seronegative cohorts.

## Introduction

Adverse and stressful events in childhood, such as parental loss, low childhood socioeconomic status, or institutionalization, have been associated with elevated levels of inflammation (1) and an increased risk for multiple age-related diseases, such as cardiovascular disease (2, 3) and type 2 diabetes (4). As many as 39% of children worldwide are estimated to experience one or more forms of early life adversity (ELA) (5), placing a high economic burden on health-care systems—and society in general—through medical costs and lost productivity (6). Although ELA is a major and global public health problem, it is currently unknown how its detrimental consequences can be prevented or reversed.

Many efforts have been made to understand the mechanisms underlying long-term effects of ELA. One of the mechanisms proposed is accelerated aging of the immune system, also known as immunosenescence (7, 8). Immunosenescence refers to the process of progressive deterioration of immune functions that go hand in hand with normal aging (9). Although senescence is characterized by an irreversible arrest in cell proliferation, senescent cells are not inactive, but show elevated levels of cytotoxicity and produce more pro-inflammatory cytokines (10). Accelerated immunosenescence negatively impacts health, leading to increased lifetime morbidity and mortality (11, 12). Thus, if ELA affects the rate of immunosenescence, this may explain an increased risk and earlier onset of age-related disorders.

Indeed, evidence is accumulating that ELA accelerates immunosenescence. One of the most used proxies for immunosenescence is telomere length. Telomeres are tandem repeats at the ends of chromosomes that shorten with age and with every cell division (13). For instance, naïve T cells have longer telomeres than terminally differentiated T cells that went through more replication cycles (14). A considerable number of studies have investigated telomere length in individuals with a history of ELA, but results vary in size and significance. Ridout et al. included 41 studies (*n* = 30,773) in a meta-analysis and could demonstrate a significant association between ELA and shorter telomeres, although with a small-to-medium effect size (15). A number of tissues were included, including buccal cells, but the majority of studies focused specifically on leukocytes.

However, leukocytes are a heterogeneous mixture of immune cells; ELA may affect some cell types more than others. Few studies have investigated immunosenescence in specific immune subtypes. To the best of our knowledge, only Cohen et al. measured telomere length in a specific immune subset. They investigated a terminally differentiated and senescent subset of T cells, CD8^+^CD28^−^ cells and found shorter telomeres associated with low childhood socioeconomic status (16). These data suggest that ELA specifically affects the aging of T cells, although other cell types are probably affected as well.

T cell senescence is characterized by a loss of naïve T cell populations, which are essential to combat novel antigens from infection or vaccination. At the same time, memory cell types such as effector memory (EM) and terminally differentiated T cells gradually increase during aging (17). It is possible to differentiate between naïve, central memory (CM), EM, and terminally differentiated T cell populations, using lineage markers such as CD45RA and CCR7 (18). Moreover, several cell surface markers have been identified that are either up- or downregulated as T cell senescence progresses, such as CD57, which allow for cell type-specific analysis of immunosenescence (19).

Apart from telomere length and surface molecules as biomarkers for senescence, there is emerging literature on various epigenetic indicators of cellular aging, based on an accumulation of age-related changes in DNA methylation profiles (20). Epigenetic indices have been shown to predict mortality and biological age independently from telomere length, suggesting that epigenetic aging targets an alternative pathway to telomere length (21). Furthermore, epigenetic aging signatures have been shown to predict age more precisely than telomere length (22). The association between ELA, telomere shortening, and age-related diseases suggests that also the “epigenetic clock” ticks faster in ELA. Early results imply that psychological factors and early environment can predict epigenetic aging (23–25). However, to date, few studies have addressed this in ELA specifically, and results are ambiguous (8).

It remains an open question as to what drives ELA-associated immunosenescence. Besides ELA, several other environmental factors have been found to modulate the rate of immunosenescence, such as persistent viral infections (26). Herpes simplex virus (HSV), Epstein–Barr virus (EBV), and cytomegalovirus (CMV) are among the most prevalent viral infections that establish latency after primary infection and reactivate when the immune system is compromised. Latent infections with CMV in particular are believed to play an important role in immunosenescence and are associated with age-related alterations of T cell immunity (27, 28). Moreover, ELA increases the risk of herpes infections and has been implicated in increased reactivation in children and adults (29–31).

In this study, we investigated T cell-specific immunosenescence (T cell differentiation and CD57 expression) in participants with and without a history of ELA, in addition to epigenetic aging at age-related CpGs. Participants in the ELA group had experienced separation from their parents in early childhood and were subsequently adopted, which is a standard model of ELA. This study cohort is a healthy subset of the EpiPath cohort, excluding all participants with acute or chronic diseases. With a mediation analysis we examined whether CMV titers may account for immunosenescence observed in ELA.

## Materials and Methods

### Participants

Healthy participants were selected from the EpiPath cohort (Elwenspoek et al., manuscript under review, *Journal of Immunology*), based on absence of chronic or acute diseases and medication use, and adequate number of bio-banked peripheral blood mononuclear cells (PBMCs) for investigation. The EpiPath cohort was recruited between 2014 and 2016 from Luxembourg and The Greater Region Saar-Lor-Lux and consisted of young adults, aged 18–35 years, that were either parent-reared (Ctrl) or experienced separation from their parents in early childhood followed by adoption (ELA). 59 Ctrl and 18 ELA participants were included in this study (Figure S1 in Supplementary Material). One adoptee was directly adopted from the birth family, all others experienced the additional stress of institutionalization, which is considered to be a form of social deprivation and structural neglect (32). All participants gave their written informed consent. The study design was approved by the Ethics Review Panel of University of Luxembourg (ERP, No 13-002) and the National Research Ethics Committee (CNER, No201303/10) in compliance with the Declaration of Helsinki.

### Blood Samples

Blood samples were collected in sodium heparin-coated tubes for PBMC isolation and in EDTA-coated tubes for DNA and plasma isolation. To minimize inter-individual variation, all samples were collected at the end of the morning (ca. 11:30 a.m. ±30 min); participants were asked to refrain from smoking, strenuous physical exercise, and drinking caffeinated or alcoholic beverages on the day of the clinical visit; women were either using hormonal contraceptives or were in the luteal phase of their menstrual cycle. Furthermore, participants’ age and sex were recorded. At a second visit, information about the age at adoption was obtained and the childhood trauma questionnaire (CTQ) was administered.

### CMV Titers

EDTA blood samples were centrifuged at 4°C within 15 min of blood collection and the plasma phase was collected. Plasma samples were transported on ice and stored at −80°C within 6 h until further analysis. Plasma was used in a 1:21 dilution to determine CMV IgG antibody indexes by ELISA (Calbiotech, El Cajon, CA, USA). As a control, HSV-1 and EBV IgG antibody indexes were also determined with ELISAs from the same company. The ELISAs were performed following manufacturer’s instructions, in duplicate, and read on a SpectraMax Plus 384 Microplate Reader (Molecular Devices, Berkshire, UK).

### Telomere Length and Age-Related CpGs

DNA isolation and telomere length measurements in the EpiPath were reported previously in Elwenspoek et al. (manuscript under review, *Journal of Immunology*). Methylation levels were measured at age-related CpGs in aspartoacylase (ASPA) (cg02228185), integrin alpha 2b (ITGA2B) (cg25809905), and PDE4CA (cg17861230) according to (22). In brief, unmethylated cytosine residues in each DNA sample were converted to uracil with a bisulfite treatment (EpiTect Bisulfite Kit, Qiagen, Venlo, Netherlands) and regions of interest were amplified with PCR (PyroMark PCR Kit, Qiagen) in the bisulfite-modified DNA according to the manufacturer’s protocols. PCR products were pyrosequenced on a Pyromark ID with Pyrogold reagents (Biotage, Uppsala, Sweden) and methylation levels were analyzed with Pyro Q-CpG SW (Biotage). A sample of pooled DNA was run in each batch as internal control, which was used to calculate relative methylation levels. These relative methylation levels were used for all further analyses.

### PBMC Isolation and Flow Cytometry

All cell culture products were from Lonza BioWhittaker (Versviers, Belgium), unless otherwise stated. PBMCs were isolated within 3 h of sample collection using Ficoll-Paque density gradient centrifugation. Briefly, EDTA blood was diluted in sterile 1× PBS, layered over Ficoll-Paque™ PLUS (Fisher Scientific, Erembodegem-Aalst, Belgium) in Leucosep tubes (Greiner Bio-One, Vilvoorde, Belgium), and centrifuged for 5 min at 300 *g*. PBMCs were washed twice with PBS and stored at 4.10^6^ cells/1 mL/aliquot in 80% Heat-Inactivated Fetal Bovine Serum (Gibco, Paisley, UK) and 20% DMSO (Sigma-Aldrich, Saint-Louis, CA, USA) in liquid nitrogen until analyzed.

Peripheral blood mononuclear cells were thawed quickly and rested overnight at 37°C, 5% CO_2_ in RPMI 1640 medium, with 10% Heat-Inactivated Fetal Bovine Serum, 1% penicillin/streptomycin, 1% sodium pyruvate (Gibco), 1% non-essential amino acids (Gibco), and 1% ultra-glutamine. PBMCs (1.10^7^ cells/mL) were incubated with GolgiPlug and GolgiStop (final concentrations of 2 and 1 µL/mL, respectively, BD BioSciences) for 5 h at 37°C, 5% CO_2_. All subsequent steps were performed at 4°C and protected from ambient light. PBMCs were washed twice with 1× FACS Buffer [1× PBS, 1% bovine serum albumin Cohn fraction V (Sigma-Aldrich, Saint-Louis, CA, USA), 0.1% NaN3, 2 mM EDTA (Sigma-Aldrich), pH 8.0] and stained with a LIVE/DEAD dye and antibodies against CD4, CD3, CD8, CD45RA, human leukocyte antigen–antigen D related (HLA-DR), CCR7, and CD57 (Table S1 in Supplementary Material) for 30 min. Then, PBMCs were permeabilized and fixed with BD Cytofix/Cytoperm™ (BD BioSciences, San Diego, CA, USA) for 20 min, followed by a 30 min intracellular staining of granzyme B (GraB) and perforin (Table S1 in Supplementary Material).

Thirty thousand lymphocyte events were acquired on the BD LSRFortessa (BD BioSciences) using FACSDiva (BD BioSciences, version 8.0). Data analysis was performed with FlowJo (version 10.2, Tree Star, Ashland, OR, USA) using the gating strategy presented in Figure S2 in Supplementary Material. T cell differentiation was determined by CCR7 and CD45RA expression: naïve (CCR7^+^CD45RA^+^), CM (CCR7^+^CD45RA^−^), EM (CCR7^−^CD45RA^−^), and terminally differentiated cells (terminally differentiated effector memory; TEMRA, CCR7^−^CD45RA^+^). Relative numbers of cells (e.g., CD57^+^ cells) and median fluorescent intensity (MFI) were analyzed.

### Statistical Analysis

Group differences in telomere length, relative methylation levels of age-related CpGs, CMV titers, CTQ sum scores, and age at adoption were investigated with a Wilcoxon rank sum test with continuity correction. In the initial analysis, we constructed linear regression models to investigate group differences in cell types (flow cytometry data), in which “cell type” was included as outcome variable and both groups and experimental day were included as fixed effects; the latter to account for variation between experiments. Cell percentages were transformed with the arcsine transformation (asin(sign(x) × sqrt(abs(x)))) to stabilize variance and MFI values with a log-transformation to approximate normality. Analyses of variance (ANOVAs) were performed on each model to test the significance of the group effect. The Benjamini–Hochberg procedure was performed on all *p*-values generated by the ANOVAs to correct for multiplicity (33). When plotting CMV titers, a “high” and “low” antibody group emerged. Log(CMV titers) = −1.2 was chosen as cutoff. To investigate the relationship between CMV levels and CD57 expression, Spearman’s rank correlation rho were determined. To investigate the mediating effect of CMV on the association between ELA and senescent cells, a mediation analysis was performed in R [version 3.3.3 (34) using *mediation* version 4-4.5 (35)]. Because age at adoption can be considered to be proportional to the duration of adversity and can thus be used as proxy for ELA severity (36), in the mediation analysis, age at adoption (months) was used as continuous variable for ELA, in which controls were set to 0. *p*-Values below 5% were considered significant.

## Results

### Participant Characteristics

The Ctrl and ELA groups did not differ in age or sex. The median age at adoption was 3.4 months. Adoptions took place at an early age, so participants had no memory of the time before adoption. Consequently, the CTQ scores, based on the participant’s memory of trauma experiences before age 16, reflects experiences after adoption, which was similar between groups. Thus, apart from the adoption, the experimental groups were comparable in age, sex, subsequent childhood trauma exposure, and all participants were in good health (Table 1; Figure S1 in Supplementary Material).

**Table 1:** Participant characteristics.

	All (*n* = 77)	Ctrl (*n* = 59)	ELA (*n* = 18)	*p*-Value
Age (median years [IQR])	22 [20–24]	21 [20–23]	23 [20–25]	0.702
Sex (% female)	61.0	59.3	66.7	0.777
Age at adoption^a^ (median months [IQR])	0 [0–0]	0 [0–0]	4.3 [0–15]	<0.001
Childhood trauma (median CTQ scores [IQR])	1.2 [1.1–1.4]	1.2 [1.1–1.4]	1.2 [1.1–1.4]	0.934

*Statistics: For continuous variables, the Wilcoxon rank sum test with continuity correction was applied and for categorical variables the Pearson’s chi-squared test with Yates’ continuity correction*.

*Ctrl, control; CTQ, childhood trauma questionnaire; ELA, early life adversity; IQR, interquartile range*.

*^a^“Age at adoption” was used as a proxy of ELA severity (36); therefore, controls were set to 0*.

### Telomere Length and Epigenetic Aging

**Figure 1:**
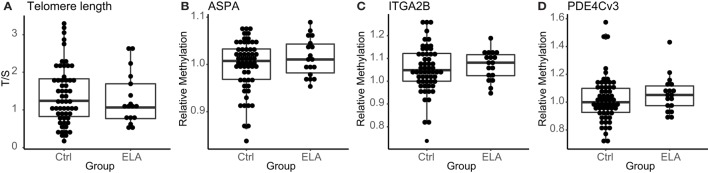
Markers for senescence. Relative telomere length in T/S **(A)**, methylation levels at the age-related CpGs ASPA, ITGA2B, and PDE4C **(B–D)**, respectively. Statistics: Wilcoxon rank sum test with continuity correction. Abbreviations: Ctrl, control; ASPA, aspartoacylase; ELA, early life adversity; ITGA2B, integrin alpha 2b; PDE4C, phosphodiesterase; T/S, relative telomere to single copy gene ratio.

Immunosenescence in leukocytes was measured with two distinct techniques. First, as previously reported, we did not observe a difference in telomere length between the two groups in the complete EpiPath cohort (Elwenspoek et al., manuscript under review, *Journal of Immunology*). Also in the subset of participants used in this investigation, which only included healthy participants, there was no effect of ELA on telomere length (median [interquartile range; IQR]; Ctrl: 1.2 [0.8–1.8], ELA: 1.1 [0.8–1.7], *p* = 0.714; Figure 1A). Second, methylation levels at three age-related CpGs that have been linked to chronological and biological age were measured (22). We found similar methylation levels in ELA and Ctrl (ASPA Ctrl: 1.01 [0.97–1.03], ELA: 1.01 [0.98–1.04], *p* = 0.452; ITGA2B Ctrl: 1.05 [1.00–1.12], ELA: 1.08 [1.02–1.12], *p* = 0.438; phosphodiesterase (PDE4C) Ctrl: 0.99 [0.93–1.10], ELA: 1.05 [0.97–1.13], *p* = 0.258; Figures 1B–D).

### T Cell-Specific Senescence

**Figure 2:**
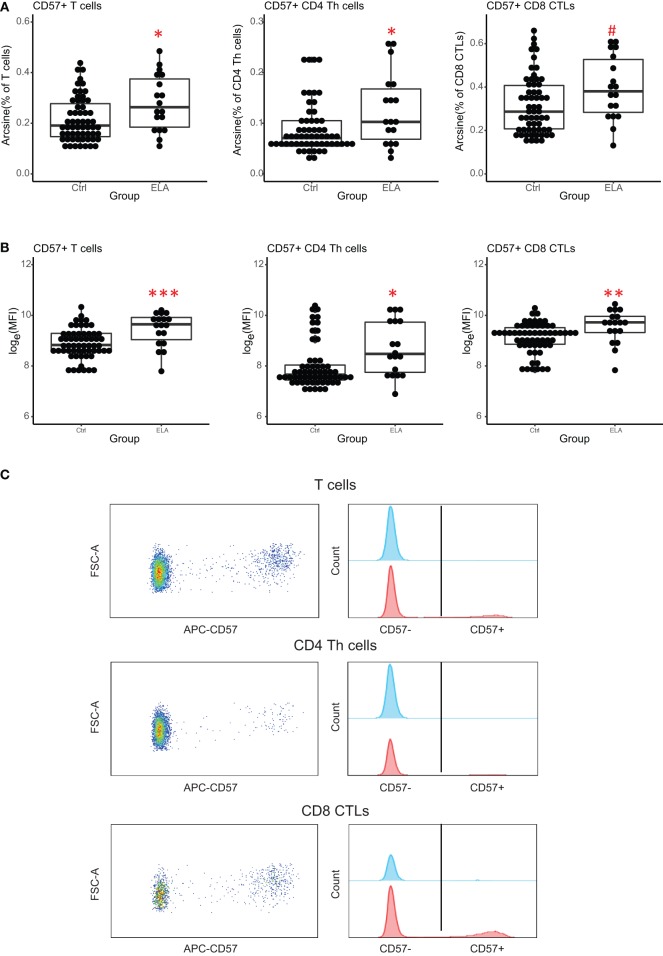
Senescence marker CD57 on T cells. ELA participants have more CD57^+^ T cells (total and subsets Th and CTLs) **(A)** and the expression of CD57 per cell is higher (MFI) **(B)**. *Y* axis shows percentages of parent populations after an arcsine transformation, or log-transformed MFI values. Representative dot plots and histograms of CD57 expression on T cells and its subsets **(C)**. Statistics: linear model with group and experimental day as independent variables, and arcsine-transformed percentages or log-transformed MFI of CD57^+^ cells as dependent variable. *p*-Values are corrected for multiplicity with false discovery rate. ^#^*p* < 0.10, *p < 0.05, **p < 0.01, ***p < 0.001. Abbreviations: Ctrl, control; ELA, early life adversity; MFI, median fluorescent intensity; CTL, cytotoxic T lymphocyte; Th cell, T helper cell.

A 10-color flow cytometry panel was used to investigate cell-specific immunosenescence. Although immunosenescence is related to changes in the ratio of naïve and memory T cells, the ratios and numbers of naïve, CM, EM, and terminally differentiated T cells (TEMRA) were similar between Ctrl and ELA (data not shown). However, we found a higher number of T cells expressing the senescence marker CD57. ELA was associated with a significant increase in both the total number of T cells (linear regression, adjusted *p* = 0.017) and T helper (Th) cell subset (adjusted *p* = 0.038), expressing CD57 (Figure 2A). The cytotoxic T lymphocytes (CTLs) showed a similar trend toward higher CD57, albeit this did not reach statistical significance (adjusted *p* = 0.061). The increase in CD57^+^ cells between Ctrl and ELA appeared to be highest in Th cells, showing almost a 1.5-fold increase, and lowest in overall lymphocytes, consisting of a mixture of B cells, T cells, and NK cells.

**Figure 3:**
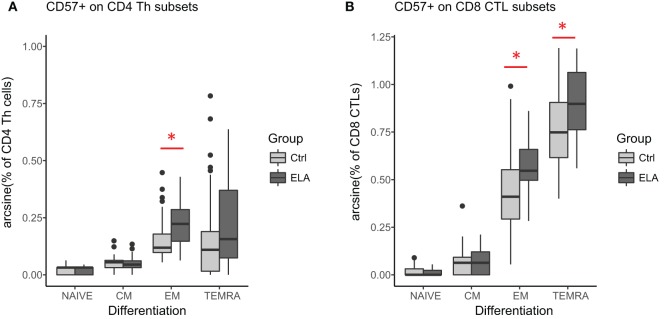
CD57 expression in different stages of T cell differentiation in CD4 Th cells **(A)** and CD8 CTLs **(B)**. Statistics: linear model with group and experimental day as independent variables, and arcsine-transformed percentages of CD57^+^ cells as dependent variable. *p*-Values are corrected for multiplicity with false discovery rate. *p < 0.05, **p < 0.01, ***p < 0.001. Abbreviations: Ctrl, control; CM, central memory; ELA, early life adversity; EM, effector memory; CTL, cytotoxic T lymphocyte; TEMRA, terminally differentiated effector memory; Th cell, T helper cell.

There was also a significant group effect on the intensity of CD57 fluorescence (MFI, T cells: adjusted *p* < 0.001, Th cells: *p* = 0.024, CTLs: *p* = 0.001; Figures 2B,C), suggesting that CD57 expression was higher in ELA. The median fluorescent intensity (MFI) of CD57 was 5–10% higher in ELA than Ctrl on lymphocytes, total T cells, Th, and CTLs. CTLs showed the lowest increase in CD57 expression. As expected (37), CD57^+^ cells were not equally distributed among the different stages of T cell differentiation (Figure 3). In both Th and CTL subsets, EM and TEMRA cells had the highest number of CD57^+^ cells. The increase in CD57^+^ cells in ELA was mainly happening in EM Th cells (Figure 3A) and in both TEMRA and EM CTLs (Figure 3B).

### Cytolytic Potential and Activation

**Figure 4:**
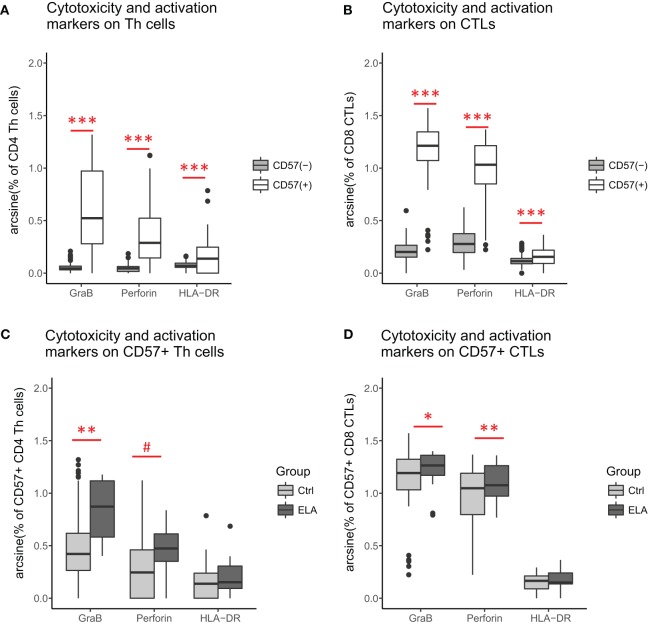
Cytolytic potential and activation status: CD57^+^ versus CD57^−^ Th cells **(A)** and CTLs **(B)**. ELA versus controls among CD57^+^ Th cells **(C)** and CTLs **(D)**. Statistics: Paired Wilcoxon rank sum test with continuity correction **(A,B)**, linear regression with group and experimental day as fixed effects, and percentage of cells as outcome variable **(C,D)**. *p*-Values are corrected for multiplicity with false discovery rate. ^#^*p* < 0.10, *p < 0.05, **p < 0.01, ***p < 0.001. Abbreviations: Ctrl, control; ELA, early life adversity; GraB, granzyme B; HLA-DR, human leukocyte antigen–antigen D related; CTL, cytotoxic T lymphocyte; Th cell, T helper cell.

Cytolytic potential was measured by GraB and perforin staining. As expected (38), CD57^+^ cells had higher GraB, perforin, and HLA-DR expression (Figures 4A,B), suggesting higher cytolytic potential and a higher activation status in senescent Th and CTLs. When comparing the senescent cells (CD57^+^) between groups, GraB and perforin expression was elevated in ELA, although the expression of the activation marker HLA-DR was similar (Figures 4C,D). The level of fluorescent intensity of neither GraB nor perforin differed between the groups (data not shown).

### Influence of CMV Infection

**Figure 5:**
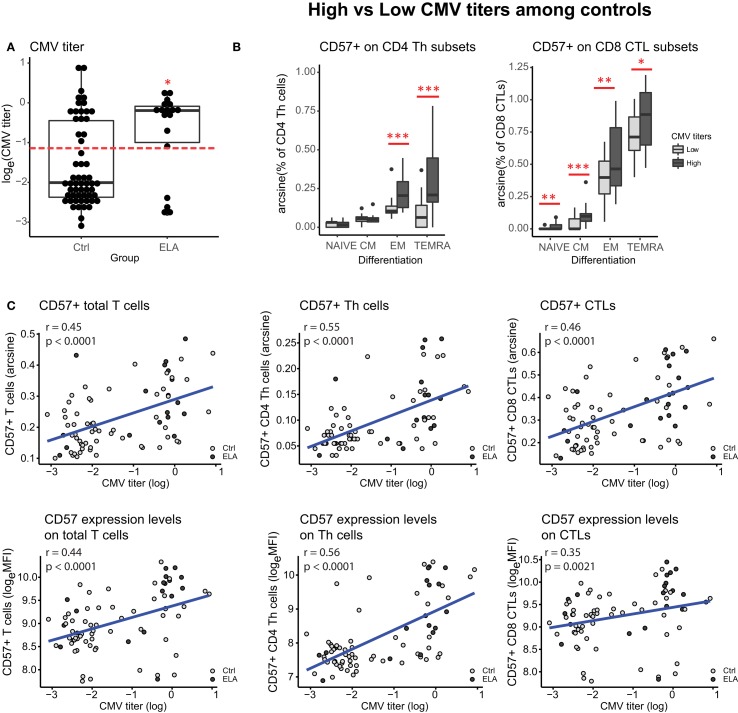
Influence of CMV infection. CMV titers in control and ELA participants **(A)**. Dotted line in panel **(A)** represents the cutoff between “high” and “low” titers used in panel **(B)**. CD57 expression in subsets of CD4 Th cells and CD8 CTLs within the control group, separated on “high” (log(CMV) > −1.2) versus “low” (log(CMV) < −1.2) CMV titers. Correlations between CMV titers and CD57^+^ numbers and expression levels on T cells (total, and subsets Th and CTLs) **(C)**. Statistics: CMV titers **(A)** Wilcoxon rank sun test with continuity correction; linear model with CMV titers (high/low) and experimental day as independent variables, and arcsine-transformed percentages of CD57^+^ cells as dependent variable **(B)**; Spearman’s rank correlation rho **(C)**. ^#^*p* < 0.10, *p < 0.05, **p < 0.01, ***p < 0.001. Abbreviations: CMV, cytomegalovirus; Ctrl, control; ELA, early life adversity; MFI, median fluorescent intensity; CTL, cytotoxic T lymphocyte; Th cell, T helper cell.

**Figure 6:**
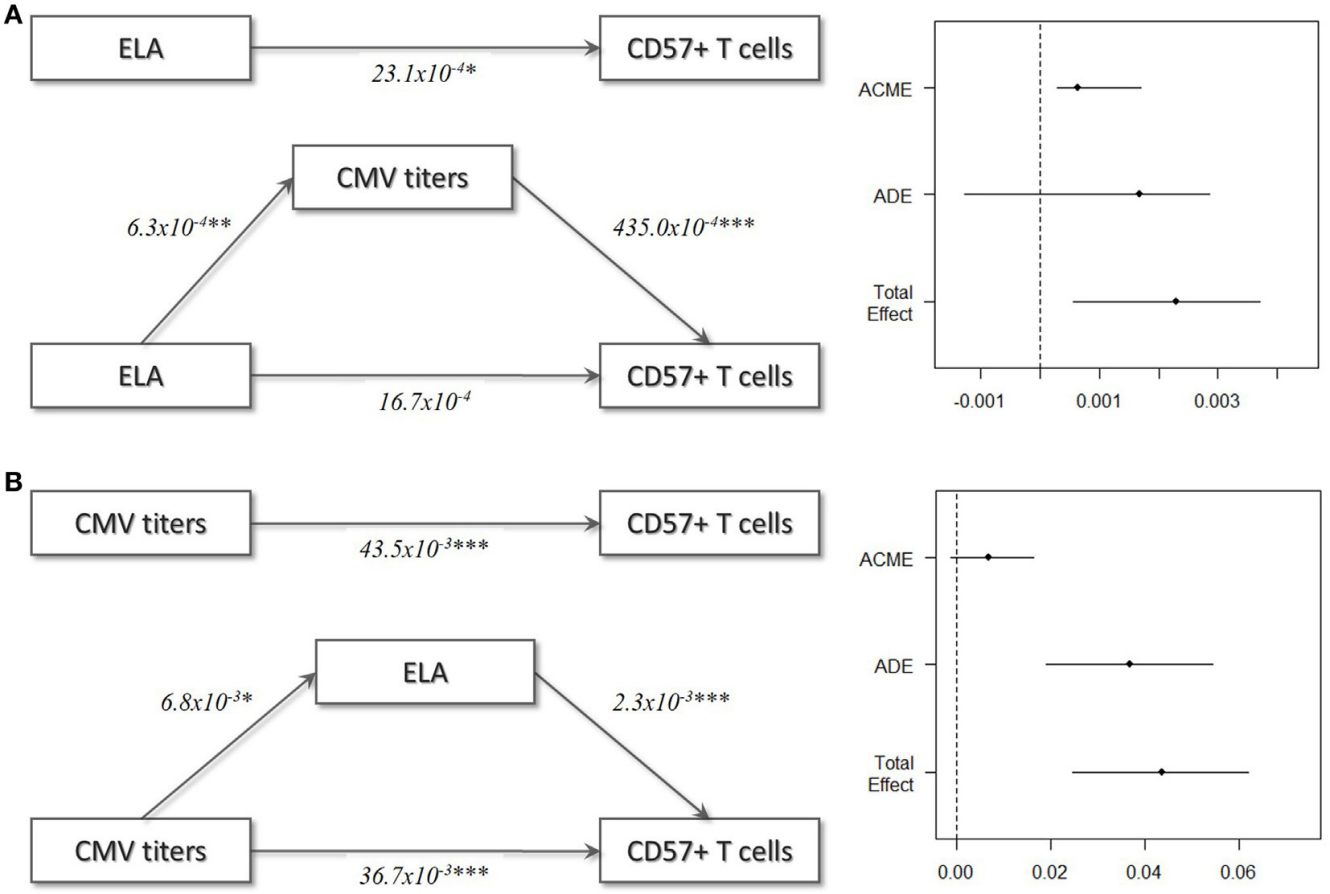
Mediation models. **(A)** ELA as independent variable, T cell immunosenescence as dependent variable, and CMV titers as mediator variable. **(B)** CMV titers as independent variable, T cell immunosenescence as dependent variable, and ELA as mediator variable. In these models, ELA was treated as continuous variable (age at adoption) and all Ctrl were set to 0. Abbreviations: ACME, average causal mediation effect; ADE, average direct effect, CMV, cytomegalovirus; ELA, early life adversity.

In the final step of the analysis, we investigated whether CMV infection had a mediating effect on the relationship between ELA and immunosenescence. Initially, we tested the difference in CMV titers between the two groups. Indeed, titers were higher in the ELA group (medium [IQR], Ctrl: 0.13 [0.09–0.64], ELA: 0.82 [0.33–0.94]; *p* = 0.023; Figure 5A), caused by an increased number of seropositive participants. To further investigate the relationship between CMV titers and CD57 expression, we divided the control participants into two groups with either “high” or “low” CMV titers. Participants with high CMV titers had significantly higher levels of CD57^+^CD4 EM (*p* < 0.001) and TEMRA (*p* < 0.001) cells, as well as higher CD57^+^ in all subsets of CD8 (Naïve, *p* = 0.007; CM, *p* < 0.001; EM, *p* = 0.001; TEMRA, *p* = 0.020; Figure 5B). Subsequently, we tested the correlation between the number of senescent cells (CD57^+^) or the level of CD57 expression on these cells and CMV titers. Indeed, we found highly significant and strong correlations in T cells, and its subsets Th and CTLs (Figure 5C). In contrast, titers of EBV and HSV, herpes viruses that cause similar lifelong latent infections, were not elevated in ELA (EBV: Ctrl: 0.91 [0.53–1.78], ELA: 1.43 [0.72–2.10], *p* = 0.213; HSV: Ctrl: 0.22 [0.12–1.78], ELA: 0.29 [0.13–1.82], *p* = 0.906) nor where they correlated to the number of senescent T cells (EBV: rho = 0.10, *p* = 0.370; HSV: rho = −0.01, *p* = 0.900). A causal mediation analysis demonstrated a large mediating effect of CMV titers (Figure 6A), which could explain 27.4% of the total effect of ELA (age at adoption) on T cell senescence (Table 2). Interestingly, when investigating ELA as mediator of the effect of CMV on senescent T cells, we only found a trend (Table 3; Figure 6B). Furthermore, there was a clear correlation between the age of adoption and CMV titer (Figure S3 in Supplementary Material, Spearman’s rho = 0.71, *p* = 0.0071), while there was no clear correlation between CMV titers and age at time of study participation among control participants (Figure S4 in Supplementary Material, Spearman’s rho = 0.17, *p* = 0.15). This suggests that ELA is not just a marker for CMV exposure, but that there are other ELA-related factors involved (Table 3; Figure 6B).

**Table 2:** Causal mediation analysis: cytomegalovirus as mediator.

	Estimate	95% CI lower	95% CI upper	*p*-Value
ACME	0.000633	0.000315	0.002202	0.00**
ADE	0.001674	−0.001614	0.002890	0.07
Total effect	0.002307	0.000586	0.003754	0.03*
Prop. mediated	0.274381	0.099298	0.661469	0.03*

Non-parametric bootstrap CIs with the percentile method. *p < 0.05; **p < 0.01.

*ACME, average causal mediation effect; ADE, average direct effect; CI, confidence interval; prop., probability*.

**Table 3:** Causal mediation analysis: early life adversity as mediator.

	Estimate	95% CI lower	95% CI upper	*p*-Value
ACME	0.00681	−0.00105	0.01637	0.08
ADE	0.03673	0.01905	0.05431	0.00***
Total effect	0.04354	0.02472	0.06183	0.00***
Prop. mediated	0.15635	−0.02606	0.38365	0.08

Non-parametric bootstrap CIs with the percentile method. ***p < 0.001.

*ACME, average causal mediation effect; ADE, average direct effect; CI, confidence interval; prop., probability*.

## Discussion

In this study, we have shown that ELA is associated with higher levels of T cell senescence in healthy participants (selection of EpiPath cohort). Even though there was no difference in telomere length or methylation levels at age-related CpGs in leukocytes, we observed a significant difference when specifically investigating T cells. Not only did we find a higher number of senescent cells (CD57^+^), these cells also expressed higher levels of CD57, a cell surface marker for senescence, and were more cytotoxic in ELA compared to controls. The difference was highest in cells in later stages of differentiation, such as EM and TEMRA cells, while differentiation *per se* was not altered in ELA (according to lineage markers CD45RA and CCR7). Control participants with “high” CMV titers showed a higher number of senescent cells, compared to controls with “low” titers, which was particularly apparent in late differentiated effector Th cells (EM and TEMRA). Importantly, we found that the effect of ELA on immunosenescence was associated with CMV infection specifically, rather than being the consequence of continued reactivation of latent viruses in general.

Our findings have important implications for this literature on senescence in ELA. Most evidence for accelerated immunosenescence in ELA comes from telomere length, but none of these studies have accounted for CMV infections. Our results suggest that the association between ELA and shorter telomeres—or immunosenescence in general—may have been largely mediated by CMV infection. Although we did not observe shorter telomeres in this study nor in the complete EpiPath cohort (Elwenspoek et al., manuscript under review, *Journal of Immunology*), we did find higher numbers of CD57^+^ T cells in ELA. It has been demonstrated previously that this cell type has shorter telomeres (39). The large inter-individual variation in telomere length and the heterogeneity of leukocytes, most probably masked this effect.

Cytomegalovirus is a likely mediator between ELA and immunosenescence. First of all, because there is a clear link between CMV infection and immunosenescence. CMV infection is related to expanding populations of specific memory T cells (40, 41), and a shrinking population of naïve T cells (42), similar to what is observed in aging (17). CMV seropositivity has been shown to reduce life expectancy by almost 4 years in an elderly population, especially due to an increase in cardiovascular deaths (43). In agreement with the results presented here, CMV-specific T cell have been found to express senescence markers, such as CD57 and KLRG1 (44), but are still highly cytotoxic (40). Interestingly, CMV infection affects reactivation of other latent viruses. Reactivation of HSV increased with age, but only in CMV seropositive individuals (45). Similarly, only in CMV seropositive individuals, EBV reactivation was associated with inflammatory markers in circulation (46). Inflammation and chronic antigen exposure as a results of viral reactivation further enhances immunosenescence (10). Finally, CMV infection has been related to reduced lymphocyte telomere length (47) and is associated with decreased telomerase activity (48), an enzyme that can partially counteract telomere loss. However, these studies looked at total T cells and leukocytes, respectively, so it is unclear whether this effect is limited to CMV-specific T cells or also affects telomere biology in other immune cells.

Second, there is reason to believe that children in adverse circumstances are at higher risk for CMV infection. For instance, the likelihood of CMV infection is higher in children raised in poverty and low socioeconomic status (49, 50). Poverty is reliable predictor of more severe forms of ELA such as childhood abuse and neglect (51–53). There is no clear epidemiological data on the prevalence of infection in international adoptees, as were included in this study. However, most adopted children have been institutionalized prior to adoption, which arguably increases the risk for CMV infection, as is the case for day-care center attendance (50). This is supported by a US study that found a 45% seroprevalence of CMV in a group of 247 internationally adopted children between 1 and 2 years of age (54) —much higher compared to the 21–22% reported in a German population sample of 1- to 2-year olds (50). Indeed, we found higher CMV titers in the adoptees compared to controls.

Although our statistical analysis suggests complete mediation by CMV, it is unlikely that CMV infection alone can explain the negative health consequences related to ELA. Especially because among seropositive individuals, ELA is associated with impaired viral control and increased CMV reactivation (29, 49, 55). Furthermore, among CMV^+^ children ELA was associated with an increased percentage of senescent CTLs (CD8^+^CD28^−^CD57^+^ cells) (56). Unfortunately, our sample size did not allow for further stratification according to CMV serostatus. Thus, even though CMV infection alone has been related to immunosenescence, ELA appears to amplify this effect.

**Figure 7:**
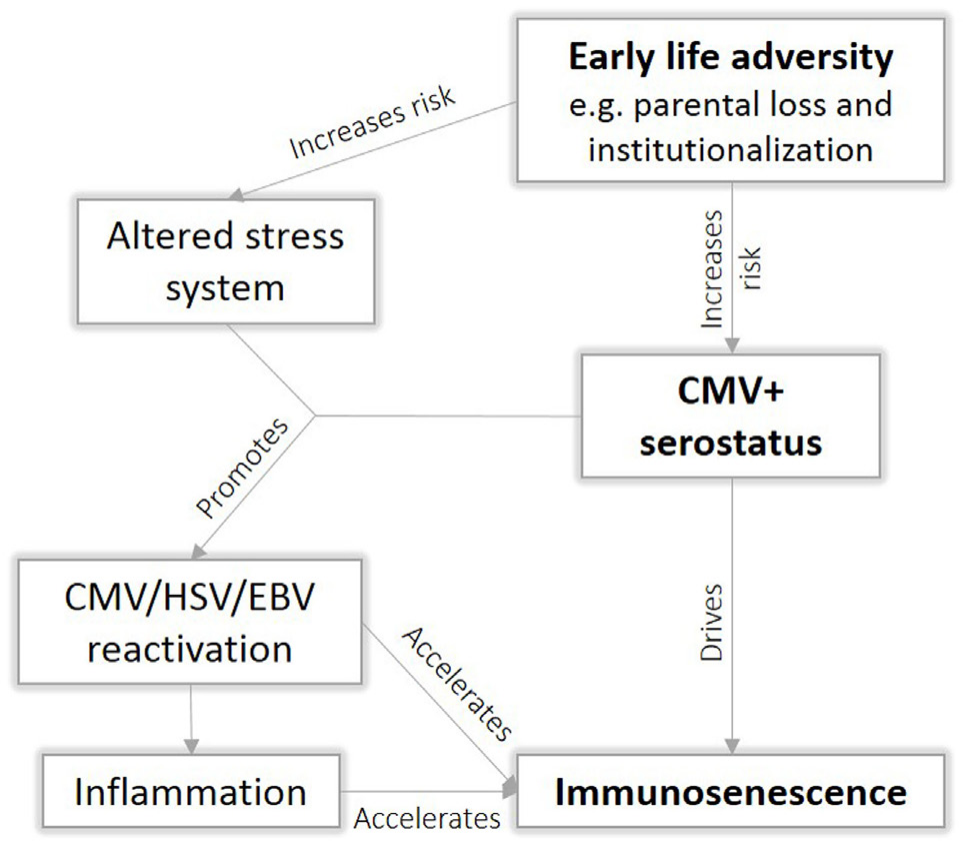
Proposed interaction between early life adversity (ELA) and cytomegalovirus (CMV) to cause immunosenescence.

Psychological stress plays an important role in viral reactivation (57), possibly due to immunosuppressive effect of the stress hormone cortisol that could compromise an effective response of the immune system. Interestingly, ELA has been association with an altered stress response (58). When we incorporate our CMV mediation model into the existing literature, it becomes clear that CMV and ELA interact on several levels, resulting in accelerated immunosenescence (Figure 7). We speculate that ELA increases the risk for CMV infection, leading to an immune response that drives T cell differentiation and thereby further accelerating immunosenescence. The effect of ELA on the stress system may subsequently compromise viral control, leading to more frequent viral reactivation, which further promotes immunosenescence. Viral reactivation results in elevated levels of inflammation, which in turn may accelerate immunosenescence *via* oxidative stress. Immune functions decline, ultimately resulting in an earlier onset of age-related diseases. However, to date, there is insufficient data to validate this hypothesis.

### Strengths and Limitations

Differences in ethnic background between the Ctrl and ELA group and small sample size were the main limitations in this study. Nevertheless, we have taken several measures to reduce variability. ELA participants came from various regions in the world, and there was not one particular region overrepresented, so we believe that a possible effect of genetic background would have been limited (Table 4). Furthermore, all blood samples were collected at the same time of day, which minimized the impact of circadian rhythms of hormones and immune cells on the results. It is important to note that in the original sample of the EpiPath cohort, ELA participants were almost four times more likely to have a chronic disease than controls. Participants with any chronic disease had higher CMV titers than healthy participants, which may have been secondary to medication use or the disease itself. To avoid effects of present disease and medication on the immune variables, we specifically selected a healthy subset of the cohort. Finally, we accounted for possible variation between experimental days by including this as a factor in the final statistical model.

**Table 4:** Countries of origin among early life adversity participants.

Country	Participants (*n*)
Brazil	1
China	2
Columbia	1
Peru	4
Romania	5
Thailand	1
Unknown^a^	4

*^a^Participant did not know or preferred not to answer*.

It is important to note that freezing and thawing as well as resting overnight of PBMCs may have introduced artifacts in the analysis. For instance, resting overnight before staining has been shown to decrease viability and can change T cell phenotype (59). Nevertheless, we could repeat our previous findings on fresh samples of the same cohort concerning HLA-DR expression on T cells (Elwenspoek et al., manuscript under review, *Journal of Immunology*).

## Conclusion

By using specific cell surface markers of senescence, we were able to detect higher levels of T cell senescence associated with ELA in a relatively heterogeneous sample of individuals. Moreover, these differences were present many years after ELA had occurred. Leukocyte telomere length may obscure cell specific immunosenescence, therefore, the use of cell surface markers of senescence or measuring telomere length on isolated cell subsets will be more informative. Although CMV appears to play an important role, it is unclear whether CMV infection is a prerequisite for ELA-related immunosenescence. To the best of our knowledge, there is no data showing an association between accelerated immunosenescence and ELA in CMV seronegative individuals. Future studies should include CMV as a confounder or selectively investigate CMV seronegative cohorts.

## Ethics Statement

In accordance with the Declaration of Helsinki, the study protocol was approved by the National Research Ethics Committee (CNER) of Luxembourg (No 201303/10 v1.4) and the Ethics Review Panel (ERP, University of Luxembourg, No 13-002). All participants provided written informed consent.

## Author Contributions

The paper was written by ME, KS, and JT, and revised into its final format by all co-authors. Participant recruitment and screening was performed by ME. Flow cytometry panel was setup by PA, ME, and KS. Flow cytometry experiments and analysis were performed by KS and ME. Plasma, PBMC, and DNA isolation were performed by SM, SS, FL, and ME. Age-related CpG methylation levels and telomere length were assayed by AE and FB. Statistical analysis was performed by ME. ME, XH, and HS were responsible for the clinical visit of EpiPah. VS and CV were responsible for the psychological visit of EpiPath. The study was conceived by CM and JT with the support and contribution of CV and HS. All authors read and approved the final manuscript.

## Conflict of Interest Statement

The authors declare that the research was conducted in the absence of any commercial or financial relationships that could be construed as a potential conflict of interest.

## Abbreviations

ANOVA, analysis of variance; CMV, cytomegalovirus; CTQ, childhood trauma questionnaire; Ctrl, control; EBV, Epstein–Barr virus; HSV-1, herpes simplex 1; IQR, interquartile range; PBMCs, peripheral blood mononuclear cells; ASPA, aspartoacylase; CM, central memory; ELA, early life adversity; EM, effector memory; GraB, granzyme B; HLA-DR, human leukocyte antigen–antigen D related; ITGA2B, integrin alpha 2b; MFI, median fluorescent intensity; PDE4C, phosphodiesterase; T/S, relative telomere to single copy gene ratio; CTL, cytotoxic T lymphocyte; TEMRA, terminally differentiated effector memory; Th cell, T helper cell.
