# Supplementary material for: T Cell Immunosenescence after Early Life Adversity: Association with Cytomegalovirus Infection
Source: Front Immunol. 2017 Oct 17;8:1263. doi: 10.3389/fimmu.2017.01263 (PMC5651086; doi:10.3389/fimmu.2017.01263)

Supplementary Material

**T cell immunosenescence after early life adversity: association with CMV infection**

**Martha M. C. Elwenspoek^1,2^, Krystel Sias^1^, Xenia Hengesch^3^, Violetta Schaan^4^, Fleur A. D. Leenen^1,2^, Philipp Adams^1^, Sophie B. Mériaux^1^, Stephanie Schmitz^1^, Fanny Bonnemberger^1^, Anouk Ewen^1^, Hartmut Schächinger^3^, Claus Vögele^4^, Claude P. Muller^1,2#^, Jonathan D. Turner^1#^*.**

*** Correspondence:** Jonathan D. Turner, PhD, [jonathan.turner@lih.lu](mailto:jonathan.turner@LIH.LU)

# Supplementary Figures and Tables

**Supplementary Table S1. Flow cytometry panel**

| **Fluorochrome** | **Ab** | **Clone** | **Company** | **Cat. N°** |
| --- | --- | --- | --- | --- |
| BUV395 | CD4 | SK3 | BDBioSciences | 563550 |
| BUV496 | CD3 | UCHT1 | BDBioSciences | 564809 |
| BUV805 | CD8 | RPA-T8 | BDBioSciences | 564912 |
| PacBlue | CD45RA | HI100 | BioLegend | 304123 |
| Bv711 | HLA-DR | G46-6 | BDBioSciences | 563083 |
| PE | GranzymeB | GB11 | BDBioSciences | 561142 |
| PE-Dazzle | CD197 | 150503 | BDBioSciences | 562381 |
| PE-Cy7 | Perforin | B-D48 | BioLegend | 353316 |
| APC | CD57 | HCD57 | BioLegend | 322314 |
| APC-Cy7 | L/D |  | LifeTech | L10119 |

**Supplementary Figure S1. Selection of participants from within the complete EpiPath cohort.**

**
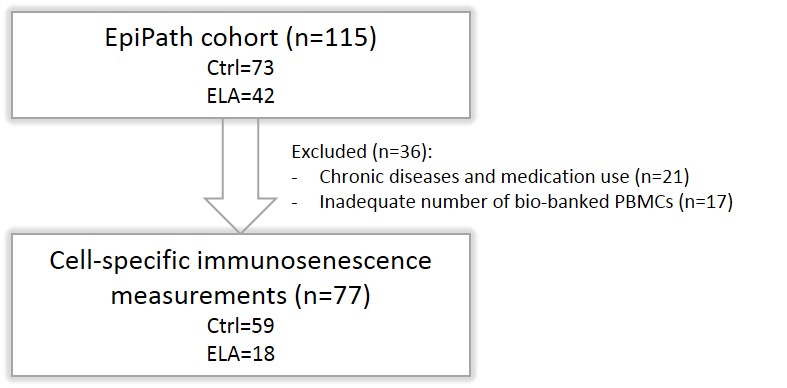
**

**Supplementary Figure S2. Gating strategy**


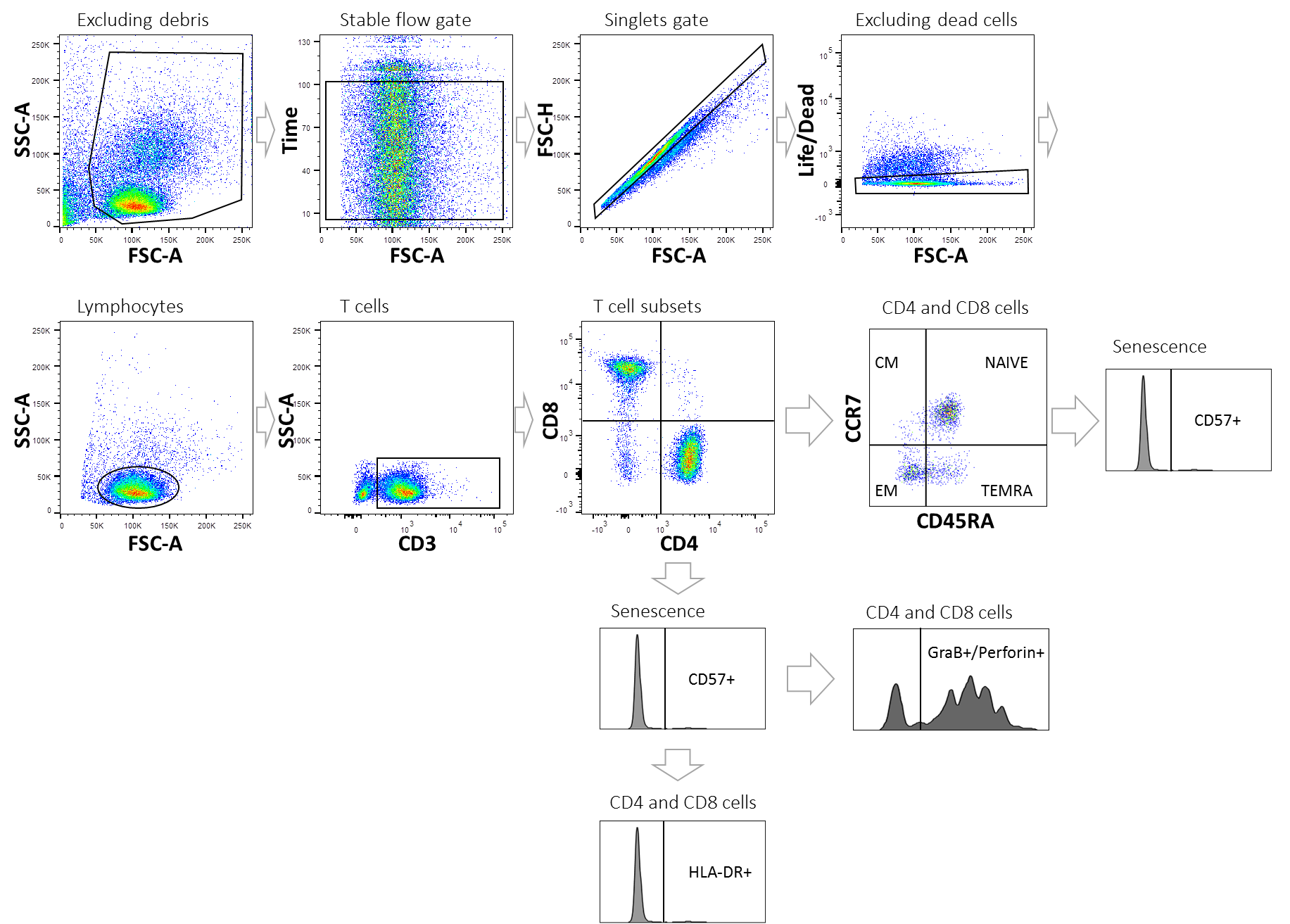


**Supplementary Figure S3**. Correlation between CMV titers and Age at adoption among ELA participants. Statistics: Spearman's rank correlation rho. Abbreviations: CMV, Cytomegalovirus; ELA, early life adversity.


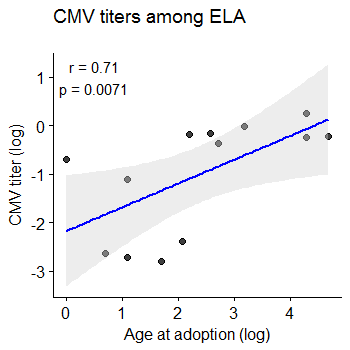


**Supplementary Figure S4**. Correlation between CMV titers and age at time of study participation among control participants. Statistics: Spearman's rank correlation rho. Abbreviations: CMV, Cytomegalovirus; Ctrl, controls.


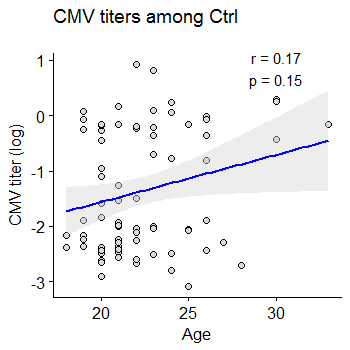

Supplement: Supplementary file 1 [file Data_Sheet_1.docx]
