# Supplementary figures and images for: T Cell Immunosenescence after Early Life Adversity: Association with Cytomegalovirus Infection
Source: Front Immunol. 2017 Oct 17;8:1263. doi: 10.3389/fimmu.2017.01263 (PMC5651086; doi:10.3389/fimmu.2017.01263)

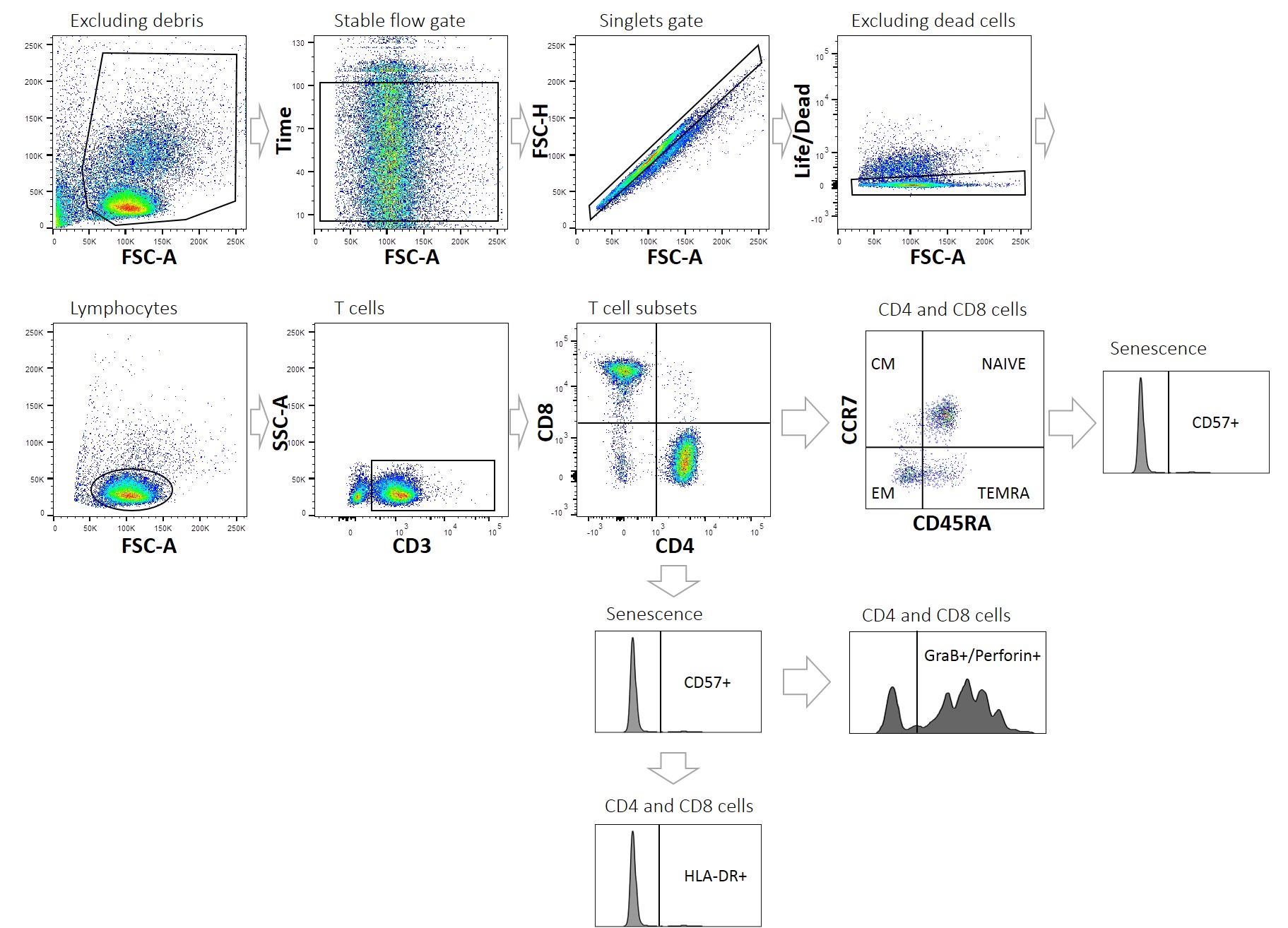

Supplement: Supplementary file 2 [file Image_1.JPEG]
